# Mapping of a novel clubroot resistance QTL using ddRAD-seq in Chinese cabbage (*Brassica rapa* L.)

**DOI:** 10.1186/s12870-018-1615-8

**Published:** 2019-01-08

**Authors:** Rawnak Laila, Jong-In Park, Arif Hasan Khan Robin, Sathishkumar Natarajan, Harshavardhanan Vijayakumar, Kenta Shirasawa, Sachiko Isobe, Hoy-Taek Kim, Ill-Sup Nou

**Affiliations:** 1 Department of Horticulture, Sunchon National University, Suncheon, 57922 Republic of Korea; 2 Department of Genetics and Plant Breeding, Bangladesh Agricultural University, 2202, Mymensingh, Bangladesh; 3 Kazusa DNA Research Institute, Kisarazu, Japan

**Keywords:** Clubroot, *Plasmodiophora brassicae*, SNP, Chinese cabbage, ddRAD-seq

## Abstract

**Background:**

*Plasmodiophora brassicae* is a soil-borne plant pathogen that causes clubroot disease, which results in crop yield loss in cultivated *Brassica* species. Here, we investigated whether a quantitative trait locus (QTL) in *B. rapa* might confer resistance to a Korean *P. brassicae* pathotype isolate, Seosan. We crossed resistant and susceptible parental lines and analyzed the segregation pattern in a F_2_ population of 348 lines. We identified and mapped a novel clubroot resistance QTL using the same mapping population that included susceptible Chinese cabbage and resistant turnip lines. Forty-five resistant and 45 susceptible F_2_ lines along with their parental lines were used for double digest restriction site-associated DNA sequencing (ddRAD-seq). High resolution melting (HRM)-based validation of SNP positions was conducted to confirm the novel locus.

**Results:**

A 3:1 ratio was observed for resistant: susceptible genotypes, which is in accordance with Mendelian segregation. ddRAD-seq identified a new locus, *CRs*, on chromosome A08 that was different from the clubroot resistance (CR) locus, *Crr1*. HRM analysis validated SNP positions and constricted *CRs* region. Four out of seventeen single nucleotide polymorphisms (SNPs) positions were within a 0.8-Mb region that included three NBS-LRR candidate genes but not *Crr1*.

**Conclusion:**

The newly identified *CRs* locus is a novel clubroot resistance locus, as the cultivar Akimeki bears the previously known *Crr1* locus but remains susceptible to the Seosan isolate. These results could be exploited to develop molecular markers to detect Seosan*-*resistant genotypes and develop resistant Chinese cabbage cultivars.

**Electronic supplementary material:**

The online version of this article (10.1186/s12870-018-1615-8) contains supplementary material, which is available to authorized users.

## Background

Clubroot is a devastating disease caused by the obligate parasite *Plasmodiophora brassicae* that affects *Brassica* species worldwide leading to severe yield losses and economic damage [1]. Japan was the first Asian country to discover clubroot in the 1890s [2], although it had already been identified in the fifteenth century in Spain and soon after in other countries across Europe as well, including England in the mid-eighteenth century and in Scotland in mid-nineteenth century [3]. In Korea, clubroot was first discovered in 1928 [2, 4]. Currently, clubroot is the major threat to Chinese cabbage production in Korea and Japan. *P. brassicae* is a soil-borne, biotrophic pathogen which makes it very difficult to control clubroot disease. Cultivation of resistant cultivars is the most effective and eco-friendly approach to controlling clubroot, although many other strategies have also been proposed [5–7].

Both qualitative and quantitative patterns of clubroot disease resistance have been identified in *Brassica rapa*. Over the last two decades, a total of 14 different loci (*Crr1*, *Crr2*, *Crr3*, *Crr4*, *CRa*, *CRb*, *CRc*, *CRk*, *PbBa3.1*, *PbBa3.3*, *Rcr2*, *Rcr4*, *Rcr8* and *Rcr9*) have been identified that are believed to govern clubroot resistance (CR) in *B. rapa* [8–16]. The major gene-based CR is due to pathotype-specific reactions with *P. brassicae* [10, 11, 17–19], indicating that resistance to each isolate is controlled by one or a few resistance genes.

In Korea, *P. brassicae* field isolates can be classified into four groups based on their ability to cause clubroot disease in Korean, Japanese, and Chinese CR cultivars [19]. Among 12 isolates collected from 10 regions in Korea, five isolates are classified as pathotype 1 (Gangneung1, Gangneung2, Goesan, Jeongseon, and Hoengseong), two isolates as pathotype 2 (Daejeon and Geumsan), three isolates as pathotype 3 (Haenam1 Pyeongchang and Yeoncheon), and the remaining two isolates as pathotype 4 (Haenam2 and Seosan) [19]. Japanese researchers developed a CR Chinese cabbage cultivar called Akimeki, which is resistant to a number of *P. brassicae* strains, including all four Japanese pathotype groups, since this cultivar bears the *Crr1*, *Crr2*, and *CRb* resistance loci [20]. However, the cultivar is susceptible to two Korean pathotype group 4 field isolates: Haenam2 and Seosan [19]. Akimeki is resistant to all other pathotypes, including Japanese pathotypes 1–3, as well as ten different field isolates collected from nine locations in Korea. These results suggest that a previously unidentified CR locus that is absent in Akimeki might govern resistance to the Korean field isolates Haenam2 and Seosan. In a recent study, ribosomal DNA sequences from *P. brassicae* field isolates collected from nine locations in Korea were found to be variable in the smaller subunit, suggesting that these isolates could be functionally different in their pathogenesis [21]. It has therefore become important to develop isolate- and pathotype-specific CR lines and relevant molecular markers for detecting isolate-specific resistant lines. To develop CR genotypes resistant to pathotype 4 that includes both the Seosan and Haenam2 isolates, the first step is to identify the quantitative trait loci (QTLs) that govern resistance. Turnip (*B. rapa*) genotypes have long been used as a valuable source of CR for introgression into Chinese cabbage cultivars [5, 18, 22–25].

The objective of the current study was therefore to identify the locus governing CR from a mapping population including a Chinese cabbage line and a turnip line that are susceptible and resistant to a *P. brassicae* Korean Seosan-isolate, respectively. We used double digest restriction site-associated DNA sequencing (ddRAD-seq) in a comparatively robust F_2_ mapping population to identify genetic variations in nucleotide positions between the resistant and susceptible genotypes. Further, a high-resolution melting (HRM) analysis was conducted to validate the SNPs that had high LOD (log_l0_ of thelikelihood ratio) scores according to QTL mapping. Yu et al. [16] recently identified another clubroot resistance QTL, *Rcr9*, on chromosome A08 of *B. rapa* that provides resistance against a newly identified pathotype 5x. A PCR-based assay was conducted using a *Crr1* (Bra020861, also a candidate of *Rcr9* [16])-specific marker to confirm that the presence of the *Crr1* locus was not associated with Seosan resistance and therefore that the newly identified source of resistance (*CRs*) must represent a novel locus. Finally, a separate PCR-based assay was conducted to show that another candidate of *Rcr9* gene (Bra020936)-specific marker was unable to differentiate between Seosan-isolate resistant and susceptible F_2_ lines.

## Methods

### Collection of *Plasmodiophora brassicae* pathotypes

Infected clubroot galls were collected by Kim et al. [19] and Jo et al. [26] between 2009 and 2013. The Seosan isolate was assigned to pathotype group 4. We used the Seosan isolate to screen resistance and susceptibility of Chinese cabbage lines. The screening procedure was essentially the same as that previously described by Kim et al. [19]. A susceptible Chinese cabbage line (CC-F920) and a resistant turnip line (SCNU-T2016) were selected to produce the segregating population.

### Production of the segregating population

The resistant turnip line (SCNU-T2016) was used as a donor parent to transfer resistance-related genes to the susceptible Chinese cabbage line (CC-F920). F_1_ plants were subsequently selfed to obtain the F_2_ population.

### Spore collection from Seosan clubs

Spores were collected from frozen club root-infected galls according to the protocol described by Feng et al. [27]. A 10% (*w*/*v*) sucrose solution was used to homogenize galls with a mortar and pestle. The liquid mixture of sucrose solution with spores was passed through an eight-layered cheesecloth. The suspension was centrifuged at 50×g for 5 min. The supernatant was separated and centrifuged at 2000×g for 5 min. The second centrifugation process produced two layers: the upper layer containing spores with a white to brownish appearance was distinctly separated from supernatant. This upper layer was initially suspended in 5 mL of double-distilled water with gentle pipetting and then more water was added to a final volume of 40. The spore concentration was adjusted to 1 × 10^7^ mL^− 1^.

### Procedure for infecting Chinese cabbage with the Seosan isolate and scoring disease

**Fig. 1:**
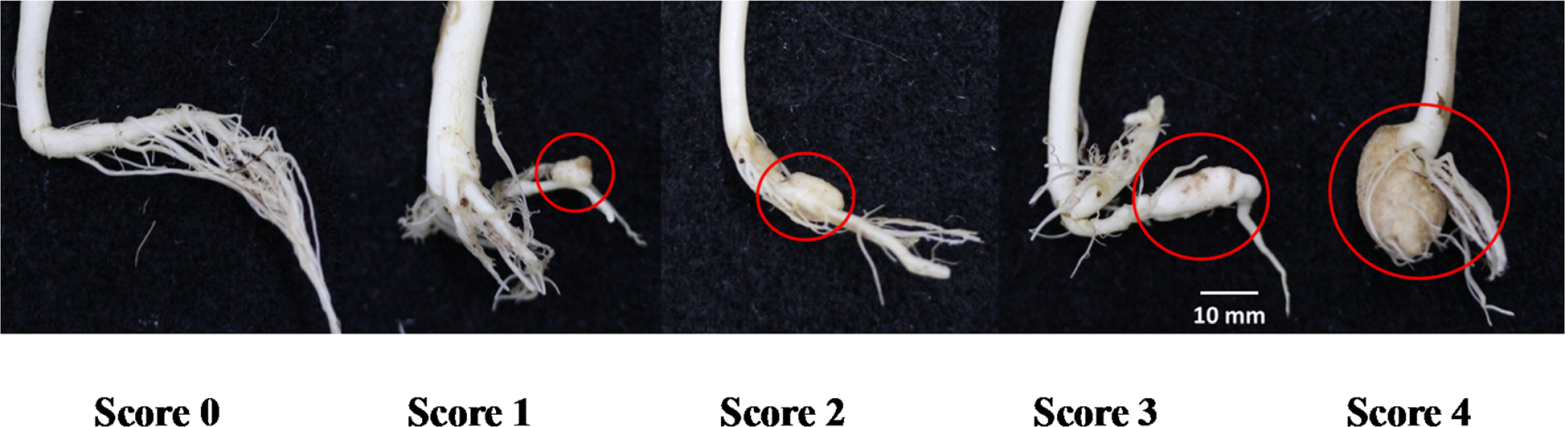
Clubroot disease-infected and non-infected roots at 45 days after sowing and 35 days after infection. A disease index (DI) score on a scale from 0 to 4 shows the severity of infection in 348 F_2_ lines. DI 0 represents no infection. DI from 1 to 4 represents clubroot infection with gradually increasing disease severity. Red circles highlight the infected region

Seedlings from parental, F_1_, and F_2_ lines were inoculated with concentrated spore suspension (1 × 10^7^ mL^− 1^) of the Seosan isolate at 10 days after sowing. There were three replicates for each line. About 5.0 mm root tip was excised from each seedling and roots were dipped into 5.0 mL spore solution. Each of the inoculated seedlings was transplanted into a single pot. The seedlings were grown in a plant culture room at 25 ± 1 °C and a 12 h day and 12 h night cycle. Each individual pot was covered by a perforated, transparent polythene bag to maintain high internal relative humidity. Club formation was investigated at one-week intervals. At 35 days after inoculation, infection was scored on a disease index (DI) following a modified method that uses a scale of 0–4, where 0 = no gall, 1 = a single club or a few small clubs of less than 5 mm in diameter formed on the lateral roots, 2 = a few clubs larger than 5 mm on the lateral roots or main root, 3 = a few medium size club 5–10 mm on the main root, and 4 = severe clubbing larger than 10 mm on either the main root or lateral roots (Fig. 1) [9, 19]. Plants with an average DI of 0.0 were classified as resistant, and plants with scores higher than 1.0 were classified as susceptible.

### Extraction of DNA from parental and F_2_ population lines

To extract genomic DNA (gDNA), young leaves from the F_2_ lines were collected, immediately snap frozen with liquid nitrogen, and stored at − 80 °C. Genomic DNA was extracted using the QIAGEN DNeasy plant mini kit (QIAGEN, Valencia, California, USA).

### Double digest RAD-Seq analysis

A total of 45 F_2_ lines from each of the resistant and susceptible phenotypes from a cross between the clubroot-susceptible line (CC-F8920) and the CR line (SCNU-T2016) as well as the parental lines (three replicates for each) were selected for ddRAD-seq analysis. A set of ddRAD-Seq libraries were constructed for selected 96 lines. The double digest RAD-Seq (ddRAD-Seq) analysis was executed using the procedures described by Shirasawa et al. [28]. Double digestion of 250 ng genomic DNA from each line was accomplished using two restriction enzymes PstI and MspI (Fast Digest restriction enzymes; Thermo Fisher Scientific, Waltham, MA, USA). The double-digested genomic DNA was then ligated to adapters using the Liga Fast Rapid DNA Ligation System (Promega, Madison, WI, USA), and purified using Agencourt AMPure XP (Beckman Coulter, Brea, CA, USA) to eliminate short DNA fragments (< 300 bp). Purified restriction-digested DNA was diluted with water and amplified with PCR using indexed primers (see Additional file 1). The PCR mixture (50 μL) contained 0.4 ng DNA, 0.2 μM of each indexed primer (one pair per mixture), 1× PCR buffer of KOD –plus– Ver. 2 (Toyobo, Osaka, Japan), 160 μM dNTPs, 1 mM MgSO_4_, and 1 U DNA polymerase (KOD –plus–; Toyobo). Thermal cycling conditions were as follows: a 3 min initial denaturation at 95 °C; 20 cycles of 30 s denaturation at 94 °C, 30 s annealing at 55 °C, and 60 s extension at 72 °C; and a final 3 min extension at 72 °C. Amplicons were pooled and separated on a BluePippin 1.5% agarose cassette (Sage Science, Beverly, MA, USA). Fragments of 300–900 bp were gel purified using the QIAGEN Mini Elute Kit (Qiagen).

The KAPA Library Quantification Kit (KAPA Biosystems, Wilmington, MA, USA) was used for measuring concentrations of the DNA libraries on an ABI-7900HT real-time PCR system (Life Technologies). The libraries (one for the F_2_ mapping population and another for the parental lines) with index tags distinguishing each line were pooled and sequenced on an Illumina HiSeq (Model: HiSeq2000, in paired-end, 93 bp mode), yielding an average of 2.8 million reads per line. The reagent used in sequencing was TruSeq SBS Kit v3-HS 200 Cycles (Illumina).

### Computational processing of ddRAD-Seq data

The FASTX-Toolkit (version 0.10.1, http://hannonlab.cshl.edu/fastx_toolkit) was used to remove low-quality sequences and to trim adapters by using PRINSEQ (−trim_right 1 -trim_qual_right 10 -derep) and fastx_clipper (−a AGATCGGAAGAGC -M 10 -n) programs. About 1.8 million high-quality reads were obtained for each line after removing low-quality sequences and trimming of adapters. The filtered reads were mapped onto the *B. rapa* genome sequence (IVFCAASv1, [29]) using Bowtie 2 (version 2.1.0; parameters: --minins 100 --no-mixed) (NCBI accession: SRR7772575-SRR7772670) [30]. This *B. rapa* genome database is similar to another *B. rapa* genome database, BRAD [31]. The resulting sequence alignment/map format (SAM) files were converted to binary sequence alignment/map format (BAM) files and subjected to single nucleotide polymorphism (SNP) calling using the mpileup option from SAM tools (version 0.1.19; parameters: -Duf) and the view option from BCF tools (parameters: -vcg). Furthermore, variant call format (VCF) files were filtered with VCF tools (version 0.1.11; parameters: --minQ 20--minDP 10 --max-missing 0.5 --maf 0.2 --min-alleles 2 --max-alleles 2 --remove-indels for the F_2_ data) [32], in which parents must be homozygous and different genotypes. Any missing value was attributed using Beagle4 [33].

### Linkage map construction

The linkage map was constructed using QTL Ici Mapping software version 4.1.0.0 [34]. To construct the linkage map with 4230 SNP markers, 2154 markers were randomly selected including at least 200 makers from each chromosome. Among the 2154 SNP markers, 2152 markers appropriately anchored with the 10 chromosomes in *B. rapa*. QTL mapping was conducted using Windows QTL cartographer version 2.5_011. During QTL mapping, 1000 permutations and a 0.05 significance level were applied. Both interval mapping (IM) and composite interval mapping (ICM) functions were used in Ici Mapping [34].

### Probe and primer design, and HRM validation

For designing probes and primers to validate each of the 17 SNPs with high LOD scores (over 20), 300 bp nucleotide sequences of *B. rapa* genome were retrieved from the EnsemblePlants database (IVFCAASv1) with the target SNP positioned as the 150th nucleotide. Each of the 17 probes was designed with a phosphorylated 3′ end (Additional file 2: Table S1). The probes were synthesized by Bioneer, Inc., Alamedia, CA, USA (Additional file 2: Table S1). All probes were designed to be intergenic. A total of 17 forward and reverse primer sets were also developed to amplify the designed probe sequences. These forward and reverse primers were synthesized by Oligo Macrogen, Seoul, Korea (Additional file 2: Table S1). A real-time PCR thermocycler (LightCycler 480, Roche Applied Science) was used for HRM analysis. The HRM conditions, composition of the HRM-PCR mixture, and HRM curve analyses, were done according to the protocol used by Laila et al. [21]. In brief, a PCR mixture for HRM was prepared by mixing 1 μL genomic DNA at 5 ng·μL^− 1^, 0.1 μL forward and 0.5 μL reverse primers at 10 pmol·L^− 1^, 1 μL probe, 7.4 μL ultra-pure water, and 10 μL of QuantiSpeed HRM Kit master mix (PhileKorea, Deajeon, Korea). The PCR was conducted with the following conditions: pre-incubation for 10 min at 95 °C, followed by 50 cycles of amplification including denaturation for 20 s at 95 °C, annealing for 20 s between 63 and 55 °C under touchdown command, and melting for 20 s at 72 °C. The amplified PCR products were then subjected to the following HRM cycle: 60 s at 95 °C, 120 s at 40 °C, and 1 s at 83 °C. The fluorescence value (^−dF^/_dT_) was recorded during the final step with five readings per °C. The LightCycler 96 software (Roche, Mannheim, Germany) was used to analyse the HRM curves. Each of the samples was appropriately labelled in sample editor. Both delta T_m_ discrimination and curve shape discrimination were set at 75%, whereas a 0.2 positive/negative threshold level was set to obtain the final output.

### PCR confirmation of novelty for the new resistance locus

To confirm that the newly identified locus is novel and different from the adjacent resistance locus *Crr1*, a set of 11 samples were tested with a *Crr1*-specific primer (Forward sequence: GATTACCACTATGTACTGAACT and reverse sequence: CTTTCAAAAACGATTGAAATTTCAT). The samples were Akimeki (*Crr1-*resistant, homozygous), Cheonghajanggun (*Crr1-*resistant, heterozygous), Bulam-3-ho (*Crr1-*susceptible, homozygous), Seosan-resistant parent (SCNU-T2016, two samples), Seosan-susceptible parent (CC-F920, two samples) and two samples each from the resistant and susceptible F_2_ lines (one homozygous and one heterozygous from both types). DNA from the Chinese cabbage lines, Akimeki, Cheonghajanggun and Bulam-3-ho was extracted following the protocol utilized for F_2_ lines. PCR amplification of DNA was conducted following previously described methods [35]. Similarly, a separate PCR amplification was conducted with six samples (two Akimeki, and two lines from each resistant, heterozygous-resistant and susceptible F_2_ genotypes) to show that the candidate *Rcr9* gene (Bra020936, Forward sequence: AGTGGAGAACCAAAGCCAAA and reverse sequence: CAATTGTGGCCACCTTCTTC) is not *CRs*.

## Results

### F_2_ phenotyping

Clubroot-infected and non-infected lines were separated based on disease scores. Out of the 348 F_2_ lines, 262 lines did not display any disease symptoms after inoculation with the Seosan isolate (Table 1). The remaining 86 lines displayed moderate to severe disease symptoms, which were scored using a 1.0 to 4.0 scale (Table 1). The ratio between resistant and susceptible lines was close to 3:1. In a chi-square test, the observed ratio fit well with the expected ratio 261:87 ≈ 3:1 indicating that a single dominant gene controls the resistance (Table 1). Out of the 86 lines showing disease symptoms, only 58 lines had severe symptoms in both the main root and lateral roots. The remaining 28 diseased lines exhibited clubs in either the main root or lateral roots, but not both.

**Table 1:** Frequency of F_2_ population phenotypes distributed across six different disease indices

Parents and crosses	Type	Disease indices (0–4)						Phenotypes and expected ratio (3:1)			
		0	1	2	3	4	Total	R	S	χ^2^	*p*
CC-F8920	S parent	0	0	0	4	20	24	0	24		
SCNU-T2016	R parent	24	0	0	0	0	24	24	0		
CC-F8920 × SCNU-T2016	F_1_	24	0	0	0	0	24	24	0		
CC-F8920 × SCNU-T2016	F_2_	262	12	8	8	58	348	261	87	0.015	0.91

The χ^2^ value indicates goodness of fit of resistant and susceptible phenotypes to the 3:1 ratio

### ddRAD-Seq analysis

Mapping rates for the susceptible Chinese cabbage line (CC-F8920) and the resistant turnip line (SCNU-T2016) were 68.7 and 64.6%, respectively, while that of the F_2_ population was 65.1% on average. Out of 255,706 SNP candidates, a total of 244,618 loci with a quality of < 999, depth of < 10, or heterozygous SNPs that probably resulted from sequencing and/or alignment errors, were eliminated. The remaining 11,088 loci were retained for further analysis. By allowing 50% missing data for each SNP locus across the 90 F_2_ lines, 5529 positions (depth of coverage of 13.5 on average) out of the 11,088 loci were selected as high-confidence segregating SNPs in the F_2_ population.

**Fig. 2:**
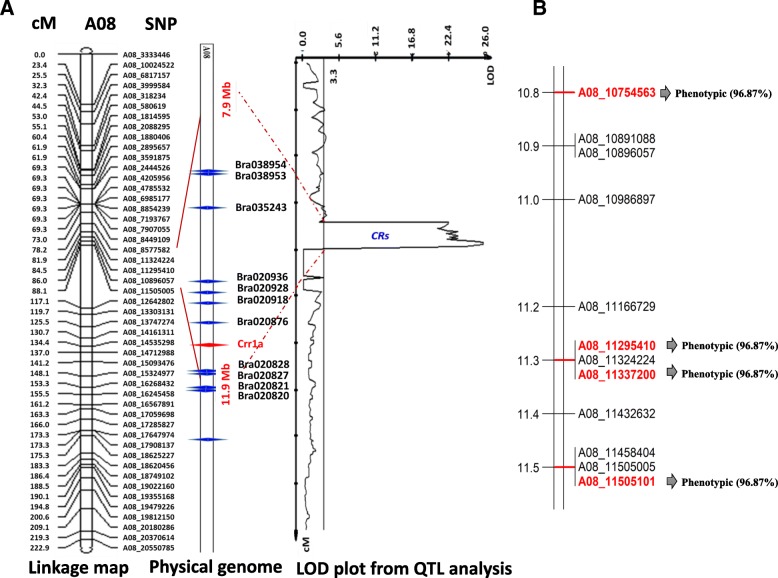
The clubroot disease resistance linkage map identified in chromosome A08 from Chinese cabbage (*Brasssica rapa* var. *pekenensis*) (**a**). The mapped locus is resistant to the pathotype 4 isolate Seosan. The SNP haplotyping was done by ddRAD-seq using a mapping population containing 45 resistant and 45 susceptible F_2_ genotypes and 3 resistant and 3 susceptible parents. ddRAD-seq was accomplished using 2152 markers. QTL mapping was done using Windows QTL cartographer with 1000 permutations and a significance level of 0.05. Four HRM probes separated resistant and susceptible members of the F_2_ population (**b**), see Additional file 6: Figure S1

Based on SNP marker separation, it was evident that the 45 resistant and 45 susceptible genotypes resulted in separable haplotypes on chromosome A08 (see Additional file 3). The ddRAD sequencing identified 390 SNPs on chromosome A08 located within 176 genes (Additional file 4: Table S2). The QTL IciMapping software mapped 54 SNP positions located in 20 genes at 0.0–222.9 centimorgans (cM) on Chinese cabbage chromosome A8 (Fig. 2, Additional file 4: Table S2). Among the 54 SNP positions, 17 SNPs yielded high LOD scores between 20.7 and 27.6. The SNP position A08:11505101 resulted in the highest LOD score of 27.758. The whole identified locus, which we named *CRs* (clubroot locus resistant to Seosan), covered 12.02 cM. In this region, 206 genes were located (see Additional file 5), including three that had NBS-LRR or disease resistance-related domains, according to the EnsemblPlants database (*B. rapa* genome, IVFCAASv1) (Table 2).

**Table 2:** Three genes identified in the *B. rapa* genome within the *CRs* region that encode leucine-rich repeats that are candidate CR genes

Chr.	Gene id	Start	End	Strand	Description
A08	Bra020918	11,205,346	11,211,166	–	Leucine-rich repeat protein kinase
A08	Bra020876	11,501,441	11,505,187	–	Leucine-rich repeat family protein

### HRM validation of SNP haplotypes with high LOD scores

Fifty SNPs were detected within the *CRs* QTL region. HRM probes were designed and tested for the 17 SNPs that had LOD scores over 20. Only 4 out of the 17 probes displayed identical genotypes for the resistant and susceptible F_2_ plants and their respective parents, which is similar to our ddRAD-seq results (i.e., A for resistant type (R), B for susceptible type (S), and H for heterozygous) for significant melting temperature differences (Additonal file 6: Figure S1). The other 13 HRM probes did not show any conspicuous separation due to genotypic variations. Probes 55 (A8:10754563), 59 (A8:11295410), 60 (A08:11337200), and 64 (A08: 11505101) clearly distinguished most of the susceptible genotypes (blue colours) from the resistant genotypes (Additional file 6: Figure S1). Each of these four probes had approximately a 6 °C difference in the melting temperature between homozygous susceptible and resistant genotypes (Additional file 6: Figure S1c-e). Most of the resistant genotypes were either homozygous (orange) or heterozygous (red). Some resistant phenotypes produced contrasting genotypes and vice versa but their genotypes always matched those obtained from ddRAD-seq. Out of the 45 resistant F_2_ lines, 14 were homozygous and 29 were heterozygous and exhibited identical genotypes to three of the resistant parents, while the other two were susceptible type. By contrast, out of the 45 susceptible F_2_ lines, 36 F_2_ were homozygous and exhibited identical genotypes with susceptible parents, while other 9 F_2_ lines were heterozygous. The four SNP positions produced identical genotypes and, similar to the ddRAD-seq results, covered 0.8 Mb from 10.75 Mb to 11.5 Mb on chromosome A08 (Fig. 1).

### PCR amplification of *Crr1* and *Rcr9* locus

**Fig. 3:**
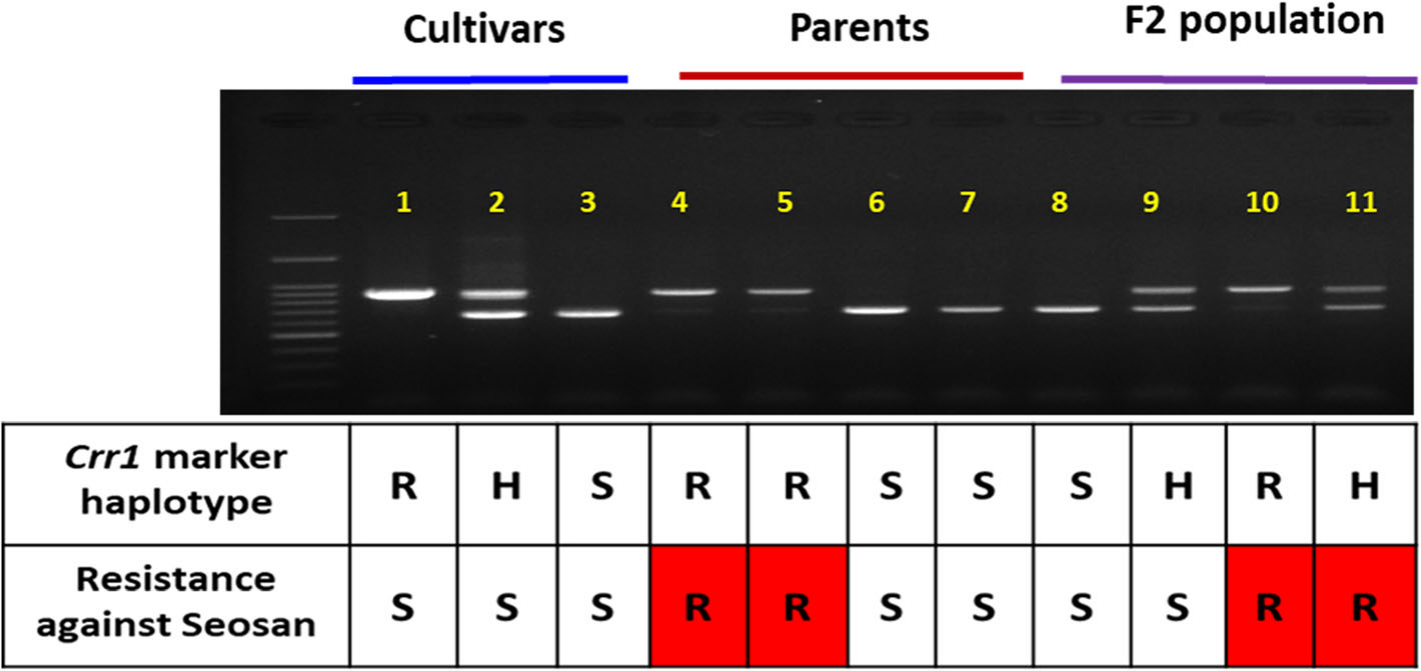
PCR amplification with *Crr1*-specific markers confirms that presence of *Crr1* gene is not enough to provide resistance against the Seosan isolate in Akimeki (Lane 1). F_2_, F_2_ population; R, resistant; S, susceptible and H, heterozygous. Sample details are 1: Akimeki, 2: Cheonhajanggun, 3: Bulam-3-ho, 4–5: Resistant parents, 6–7: Susceptible parents, 8–9: F_2_ Susceptible lines, 10–11: F2 Resistant lines

A *Crr1*-locus specific marker was used to amplify genomic DNA from resistance and susceptible F_2_ lines along with three Chinese cabbage lines Akimeki, Cheonghajanggun and Bulam-3-ho to confirm that the existence of *Crr1-*resistance haplotype had no definite association to the resistance against Seosan-isolate (Fig. 3). The Seosan-susceptible Akimeki, Seosan-resistant parent and one of the Seosan-resistant F_2_ lines produced identical amplicons (resistance-type amplicon) for the *Crr1* locus after PCR amplification (Fig. 3). The genotype Cheonghajanggun, a Seosan-susceptible F_2_ line and a Seosan-resistant F_2_ line were heterozygous for the *Crr1* locus (Fig. 3). The Seosan-susceptible parent, genotype Bulam-3-ho, and one susceptible F_2_ line produced a dissimilar and susceptible-type amplicon for the *Crr1* locus. The existence of *Crr1-*resistance haplotype in Seosan-susceptible Akimeki and heterozygous haplotype from both Seosan-susceptible F_2_ line and a Seosan-resistant F_2_ line confirmed that *Crr1-*resistance haplotype had no definite association to the resistance against Seosan-isolate. The candidate *Rcr9* (Bra020936)-specific marker resulted in identical amplicons for Akimeki, Seosan-isolate resistant and susceptible lines and thus indicated that the Bra020936 gene bearing disease resistant domain is not *CRs* (Additional file 7: Figure S2).

## Discussion

The present study sequenced a mapping F_2_ population generated between lines resistant and susceptible to the Seosan isolate of *P. brassicae* and found a dominant QTL for resistance to this pathogen. To score disease, various authors used a scale from 0 to 4 [9, 19, 36] that is similar to the one used this study. We used a modified 0–4 disease rating scale, where a rating score of 0.0 represents complete resistance and a score of 4.0 represents complete susceptibility. A large variety of gall types were observed on the susceptible parents and in the F_2_ population. Forty-five randomly selected infected F_2_ lines with disease scores from 1.0 to 4.0 were considered to be susceptible generations were sampled and used for ddRAD sequencing. This selection was conducted randomly to cover expected variation between resistant and susceptible genotypes in the F_2_ generation for ddRAD-seq. By contrast, only F_2_ lines with disease scores of 0.0 were taken as resistant F_2_ genotypes in ddRAD-seq.

ddRAD-seq is commonly used for detecting SNP variations in segregating populations [37, 38]. This technique can identify and score hundreds of thousands of SNP markers from various genotypes using Illumina next-generation sequencing with minimum resource requirements. In this study, the 17 SNPs with a LOD score higher than 20.7 had over 98.2% identical SNP haplotypes for each of the 96 genotypes including 90 F_2_ and 6 parental lines. This indicates that ddRAD-seq accurately identified a region where genotypic variation can be used to distinguish resistant and susceptible lines in the segregating population.

HRM-based genotyping analyses are becoming even more popular and reliable with the concurrent advancement of real-time PCR facilities. HRM technology can effectively and conveniently separate genotypes with only one nucleotide variation with ease. In this study, we used four HRM probes to successfully genotype 48 samples each from clubroot-resistant or-susceptible genotypes with ddRAD-seq. Among these four probes, probe 55 (A8:10754563) and probe 60 (A08:11337200) displayed a larger difference in fluorescence between genotypes compared with the two other probes, probe 59 (A8:11295410) and probe 64 (A08: 11505101), indicating that probes 55 and 60 might be more useful for genotyping Chinese cabbage lines (Additional file 6: Figure S1). Our HRM-based screening experiment successfully grouped most of the susceptible genotypes together, whereas both resistant homozygous and heterozygous genotypes were clustered into a separate group. Our phenotyping data are consistent with these HRM genotypes, which further supports a dominant nature for the *CRs* resistance locus.

Seosan is a highly virulent *P. brassicae* isolate belonging to Korean pathotype group 4 and race 5 according to William’s differential set [19, 26]. Our experiments show that when compared with 10 other isolates, this pathogen causes the formation of more galls of a larger size on the Chinese cabbage cultivar Bullam-3-ho (data not presented). An observed 3:1 ratio of resistant to susceptible genotypes in the F_2_ population indicates that the *CRs* locus is controlled by a dominant gene. On *B. rapa* chromosome A08, the *Crr1* QTL lies between 10,395,913 and 12,566,705 bp (Fig. 2) covering about 2.2 Mb of the *B. rapa* genome retrieved from the EnsemblePlants database. The region containing the *CRs* locus was delimited based on HRM data as being located between two SNP markers A8:10754563 and A08: 11505101 bp covering only 0.8 Mb of the *B. rapa* genome and not overlapping with the *Crr1* gene (A08: 11607138–11,622,943) on the same chromosome, suggesting that *CRs* and *Crr1* are two independent but adjacent loci.

## PCR confirmation of novelty for the new resistance locus

We conclude that *CRs* is a novel locus that is distinct from *Crr1*. PCR amplification for a *Crr1*-specific marker produced a similar amplicon size in both the Seosan-susceptible Akimeki and Seosan-resistant turnip parents and F_2_ lines (Fig. 3) indicating that the presence of the *Crr1* gene is not associated with resistance against Seosan. Given that Akimeki is susceptible to the Seoson isolate and lacks the *CRs* locus, in contrast with the Seosan-isolate resistant turnip parents and F_2_ lines that contain the *CRs* locus, it can be deduced that *CRs* is a novel locus, required for Seosan resistance and different from *Crr1*. Furthermore, the heterozygosity of *Crr1* had no clear association with resistance, as the cultivar Cheonhajanggun and one of the heterozygous F_2_ lines were Seosan susceptible. These results further demonstrate that *Crr1* has no association with resistance against Seosan.

Our genome browser analysis indicated that the *CRs* region confirmed by HRM did not include *Crr1* on chromosome A08. However, the *Crr1* gene and the *CRs* locus mapped in this study are adjacent CR loci, each of which confers race-specific resistance. Thus, this study identified a novel strain-specific clubroot resistance QTL located in chromosome A08 of Chinese cabbage that confers resistance against the Korean pathotype 4 Seosan isolate.

Genes bearing LRR domains, including the predicted *Crr1a*, have often been found to confer resistance against diseases [39]. Two genes, Bra020918 and Bra020876, located within the *CRs* region encode leucine-rich repeats (LRRs) and might therefore be potential candidates to underly the locus (Table 2). Identifying the specific candidate resistant genes within the *CRs* QTL region is a subject for further investigation in developing Seosan-resistant Chinese cabbage genotypes.

## Conclusion

The objective of this study was to identify the locus responsible for resistance to the *P.brassicae* Seosan isolate, which is a major clubroot pathogen in Korea. We decided to develop a QTL map to identify the probable chromosomal location. To identify the dominant gene responsible for resistance against Seosan-isolate first we need to identify the chromosomal location of that gene. Identification of the chromosomal location enable us detecting candidate genes responsible for resistance against Seosan-isolate. Thus, our QTL analysis mapped a novel dominant locus for resistance to the Seosan isolate to chromosome A08 of *B. rapa*. Genetic analysis further highlighted potential candidategenes for resistance to the Seosan isolate. The identified QTL region should be furtherstudied using PCR procedures and gene transformation to determine the generesponsible for this potentially valuable resistance.

## Additional files

Additional file 1: Indexed primers used for the PCR amplification of genomic DNA collected from parental and F_2_ lines. (XLSX 13 kb)

Additional file 2: **Table S1.** Probe numbers, identified SNP positions (red colour), probe sequences, and forward and reverse primer sequences for the validation of ddRAD-seq-identified SNPs through high resolution melting. (DOCX 33 kb)

Additional file 3: Haplotype variation between clubroot-resistant and -susceptible members of the F_2_ population obtained through ddRAD-seq. (XLSX 24 kb)

Additional file 4: **Table S2.** Number of SNPs and genes present in the whole *B. rapa* genome, chromosome 8, and the *Crr1* locus identified by ddRAD-sequencing. (DOCX 22 kb)

Additional file 5: Genes located within the 4.0 Mbp *CRs* region of *B. rapa* genome were retrieved from the EnsemblePlants database (206 genes in excel file). (XLSX 1412 kb)

Additional file 6: **Figure S1.** HRM validation of the SNP haplotypes identified through ddRAD-seq. a) Distribution of resistant and susceptible F_2_ samples in HRM. b) Heat-map showing groupings between resistant (R, A type in ddRAD-seq), susceptible (S, B type in ddRAD-seq), and heterozygous (H, H type in ddRAD-seq) genotypes. c-f) Four HRM probes that resulted in identical genotypes with ddRAD-seq. (PNG 533 kb)

Additional file 7: **Figure S2.** PCR amplification with an *Rcr9*-specific marker confirms that presence of *Bra020936* gene is unable to distinguish resistance against Seosan isolate Akimeki (Lane 1 and 2). F_2_, F_2_ population; R, resistant; S, susceptible and H, heterozygous. (PNG 286 kb)

## Abbreviations

cM: centimorgans
CR: Clubroot resistance
CRs: Clubroot resistance locus against Seosan isolate
ddRAD-seq: double digest restriction site-associated DNA sequencing
DI: Disease index
gDNA: genomic DNA
H: Heterozygous
HRM: High resolution melting
LOD score: logarithm of odds score for estimating linkage distances
LRR: Leucine-rich repeat
PCR: Polymerase chain reaction
QTL: Quantitative trait locus
R: Resistance type
RAD: Restriction site-associated DNA
S: Susceptible type
SNP: Single-nucleotide polymorphism
