# Supplementary material for: Mapping of a novel clubroot resistance QTL using ddRAD-seq in Chinese cabbage (Brassica rapa L.)
Source: BMC Plant Biol. 2019 Jan 8;19:13. doi: 10.1186/s12870-018-1615-8 (PMC6325862; doi:10.1186/s12870-018-1615-8)
Supplement: Supplementary file 2 — Table S1. Probe numbers, identified SNP positions (red colour), probe sequences, and forward and reverse primer sequences for the validation of ddRAD-seq-identified SNPs through high resolution melting. (DOCX 33 kb) [file 12870_2018_1615_MOESM2_ESM.docx]

Table S1 Probe numbers, identified SNP positions (red colour), probe sequences, and forward and reverse primer sequences for the validation of ddRAD-seq-identified SNPs through high resolution melting.

| **Probe** | **SNP Position** | **Forward primer** | **Probe** | **Reverse primer** |
| --- | --- | --- | --- | --- |
| Probe 51 | 7860590 | TTCCAAAGATAAAGCCCAGGTT | GAACCCAGCTGAGCTGCCAAG | GAAGAGGGTTTGTGGGATTGTA |
| Probe 52 | 8577582 | CCCATGATCTTTCGTACTCCTC | CAGATACTGAATATCTTTCTT | GCGAATTCCTTCATTTCACCTG |
| Probe 53 | 7313846 | GAGCGAAAACATCTTCTTGTCC | TCTAAAACTATTAATTGGTTC | CCTCACCTTCCGAGAACTAAAA |
| Probe 54 | 9067860 | GAGCGAAAACATCTTCTTGTCC | TACCATCTCCCTCGTCGTCGC | CCTCACCTTCCGAGAACTAAAA |
| Probe 55 | 10754563 | ATTCAGATGACCTTCTTCCGAC | ACTTTAGTGCGTAAGTTCTCT | GATATGTTACCACGGCGAAAAC |
| Probe 56 | 11324224 | AGAAGCTATAACATAACCTTAGCT | TTTCATGCGGCAGCGAGACTC | TGCAGATGACTACTTCCTCTCT |
| Probe 57 | 10986897 | AAAGGAGGGTGAAGAAGAAACC | CTATGTCCAACGTGTGGTAAG | TGTAGGTCTTGCTGGTTTTCTT |
| Probe 58 | 11166729 | TGATCACCTTAGCTTGTTTCGT | AGTTGAAAAGAAGTTAGAGAA | ACTGGATGATTCTTTCCTGGTT |
| Probe 59 | 11295410 | ACAGCAAAACCTCTCTTCTCTC | TTTGTCGTCGTAGCTGGTGGC | ATGTCAAACAAGCGTCTAAAGC |
| Probe 60 | 11337200 | ACACTTAGGTGAAGAAGCAACA | TTCGATGTTAGACTGCAGATA | GCATGCATCAAGTAACTTGCAA |
| Probe 61 | 10891088 | TCATGCATCACACAACCCTTAC | AATTCAAGCACGGAAAGCATG | GGAGAGAATGAAAGGGTGGTTT |
| Probe 62 | 10896057 | TTATGTCTGCAGCATCTGAACA | AATTAGAGTCGACTAAAGACA | GCTCTCCATAAAATGCTGTGTG |
| Probe 63 | 11432632 | TGTGTGGTATACTGGTATCCCA | TTTTTTTGTAGCCACAATTAA | AAAACGATCCAGGAAGATGGTA |
| Probe 64 | 11505101 | GTTGGAGAACACACTGAGATCA | CGTTCCAGGAGTTGAGGACAA | TCTCTCGAGGCTTCTGTTACTA |
| Probe 65 | 11458404 | ACAGTATGCGTTTCTGGAGAAA | AGAACGAGAAAAGAAGTGCAG | ACACAACTCTTCCACTTGAACA |
| Probe 66 | 11505005 | GAACTGGAGGCTTTTGAAACTG | AGCTCAAATTATCCAAAACAA | CTTTGTCCTCAACTCCTGGAAC |
| Probe 67 | 11863389 | TCCACCAACCATGTTGTTGATA | ACTCCTATAATTAAAAACATC | TACTTCAACAACTCGGTGGAAT |
