# Supplementary material for: Mapping of a novel clubroot resistance QTL using ddRAD-seq in Chinese cabbage (Brassica rapa L.)
Source: BMC Plant Biol. 2019 Jan 8;19:13. doi: 10.1186/s12870-018-1615-8 (PMC6325862; doi:10.1186/s12870-018-1615-8)
Supplement: Supplementary file 4 — Table S2. Number of SNPs and genes present in the whole B. rapa genome, chromosome 8, and the Crr1 locus identified by ddRAD-sequencing. (DOCX 22 kb) [file 12870_2018_1615_MOESM4_ESM.docx]

Table S2. Number of SNPs and genes present in the whole *B. rapa* genome, chromosome 8, and the *Crr1* locus identified by ddRAD-sequencing.

| **Sequencing technique** | **SNPs** | **Genes** | **Chromosome 8** | | ***CRs* locus**  **C08: 7860590–11863389** | |
| --- | --- | --- | --- | --- | --- | --- |
|  |  |  | SNPs | Genes | SNPs | Genes |
| RAD | 4,230 | 1,893 | 390 | 176 | 54 | 20 |
