# Supplementary figures and images for: Mapping of a novel clubroot resistance QTL using ddRAD-seq in Chinese cabbage (Brassica rapa L.)
Source: BMC Plant Biol. 2019 Jan 8;19:13. doi: 10.1186/s12870-018-1615-8 (PMC6325862; doi:10.1186/s12870-018-1615-8)

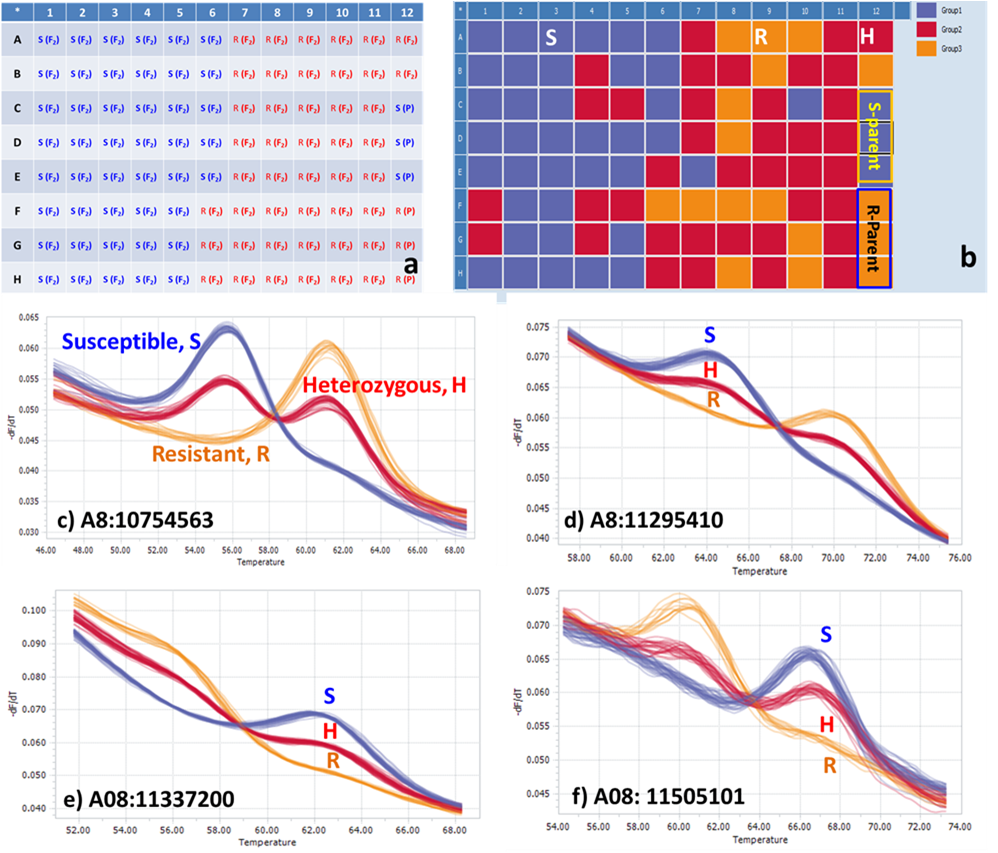

Supplement: Supplementary file 6 — Figure S1. HRM validation of the SNP haplotypes identified through ddRAD-seq. a) Distribution of resistant and susceptible F2 samples in HRM. b) Heat-map showing groupings between resistant (R, A type in ddRAD-seq), susceptible (S, B type in ddRAD-seq), and heterozygous (H, H type in ddRAD-seq) genotypes. c-f) Four HRM probes that resulted in identical genotypes with ddRAD-seq. (PNG 533 kb) [file 12870_2018_1615_MOESM6_ESM.png]

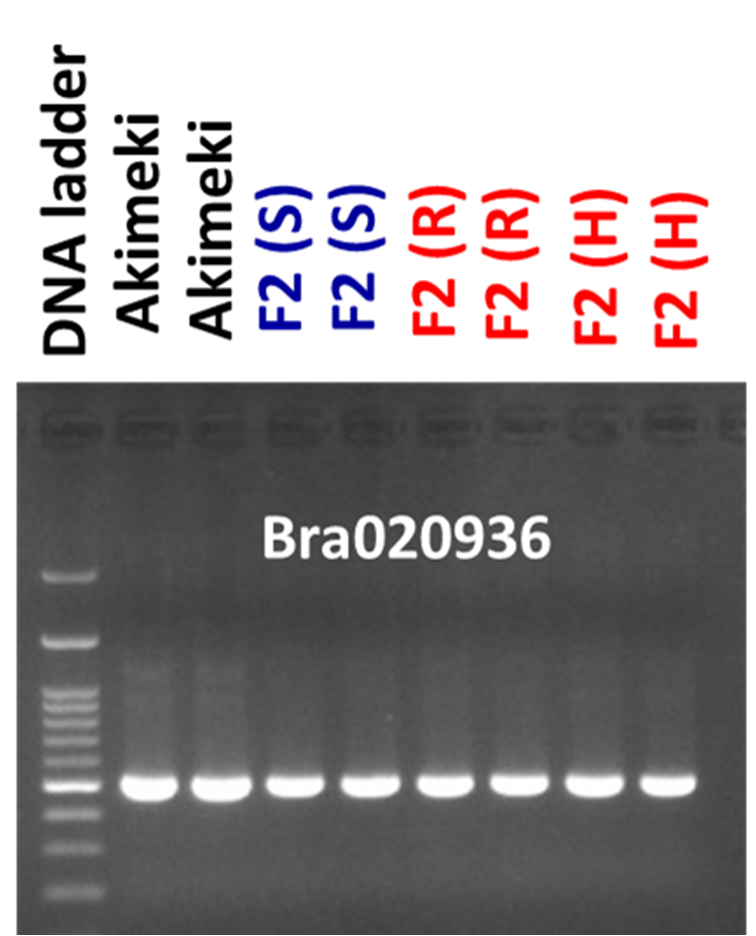

Supplement: Supplementary file 7 — Figure S2. PCR amplification with an Rcr9-specific marker confirms that presence of Bra020936 gene is unable to distinguish resistance against Seosan isolate Akimeki (Lane 1 and 2). F2, F2 population; R, resistant; S, susceptible and H, heterozygous. (PNG 286 kb) [file 12870_2018_1615_MOESM7_ESM.png]
